# Correction: Practical considerations for measuring the effective reproductive number, *R*_*t*_

**DOI:** 10.1371/journal.pcbi.1009679

**Published:** 2021-12-08

**Authors:** Katelyn M. Gostic, Lauren McGough, Edward B. Baskerville, Sam Abbott, Keya Joshi, Christine Tedijanto, Rebecca Kahn, Rene Niehus, James A. Hay, Pablo M. De Salazar, Joel Hellewell, Sophie Meakin, James D. Munday, Nikos I. Bosse, Katharine Sherrat, Robin N. Thompson, Laura F. White, Jana S. Huisman, Jérémie Scire, Sebastian Bonhoeffer, Tanja Stadler, Jacco Wallinga, Sebastian Funk, Marc Lipsitch, Sarah Cobey

There is an error in the caption for Fig 1, “Instantaneous reproductive number as estimated by the method of Cori et al. vs. cohort reproductive number estimated by Wallinga and Teunis” sentence four. Please see the complete, correct Fig 1 caption here.

**Fig 1:**
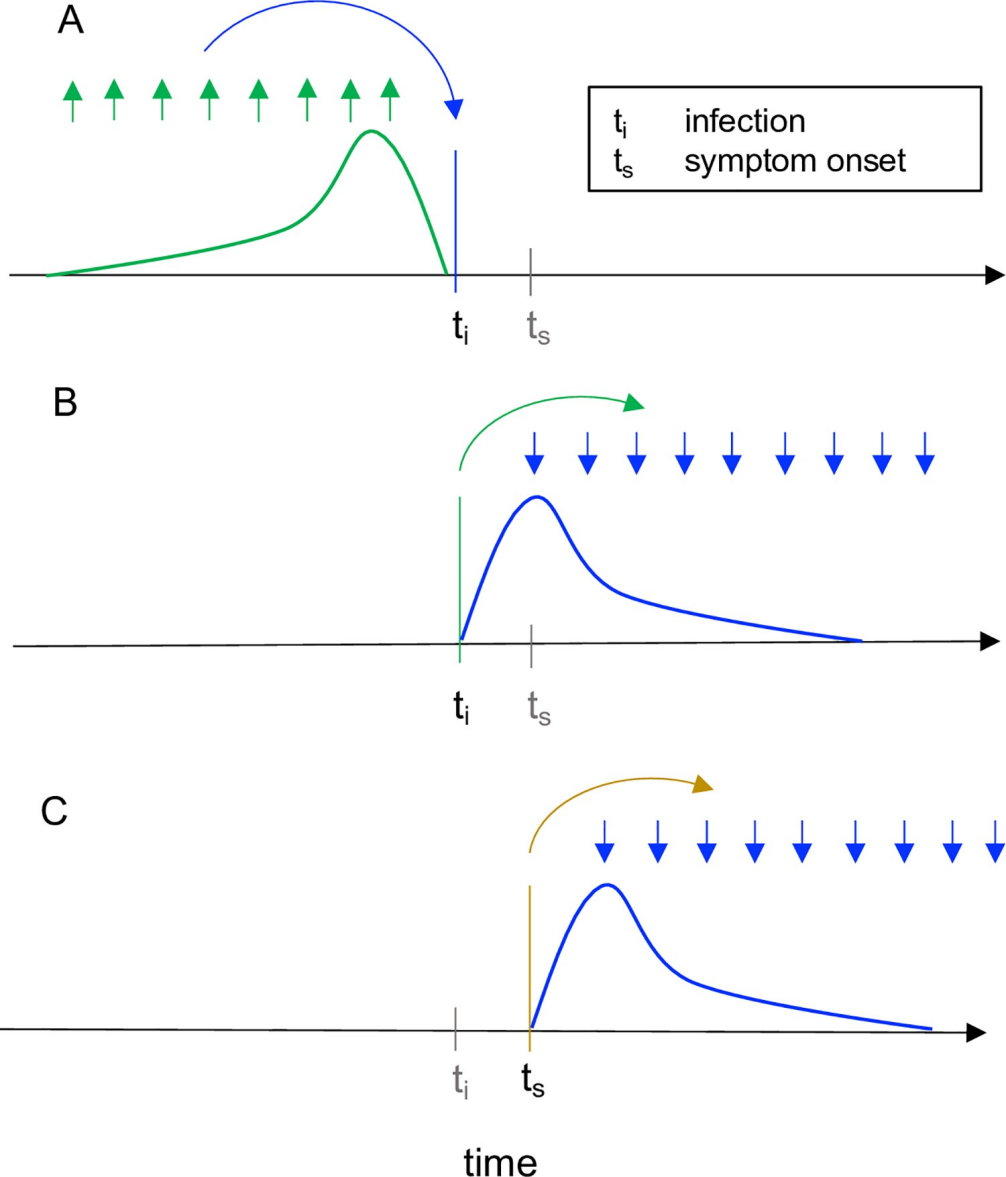
Instantaneous reproductive number as estimated by the method of Cori et al. vs. cohort reproductive number estimated by Wallinga and Teunis. For each definition of *R*_*t*_, arrows show the times at which infectors (upwards) and their infectees (downwards) appear in the data. Curves show the generation interval distribution (A, B), or serial interval distribution (C). (A) The instantaneous reproductive number quantifies the number of new infections incident at a single point in time (*t*_i_, blue arrow), relative to the number of infections in the previous generation (green arrows) and their current infectiousness (green curve). The methods of Cori et al. and of Bettencourt and Ribeiro estimate the instantaneous reproductive number. This figure illustrates the Cori method. (B-C) The case reproductive number is defined as the average number of new infections that an individual who becomes infected on day *t*_i_ (green arrows in B) or symptomatic on day *t*_s_ (yellow arrows in C) will eventually go on to cause (blue downward arrows in B and C). The first definition applies when estimating the case reproductive number using inferred times of infection, and the second applies when using data on times of symptom onset. The method of Wallinga and Teunis estimates the case reproductive number.
