# Supplementary figures and images for: Practical considerations for measuring the effective reproductive number, Rt
Source: PLoS Comput Biol. 2020 Dec 10;16(12):e1008409. doi: 10.1371/journal.pcbi.1008409 (PMC7728287; doi:10.1371/journal.pcbi.1008409)

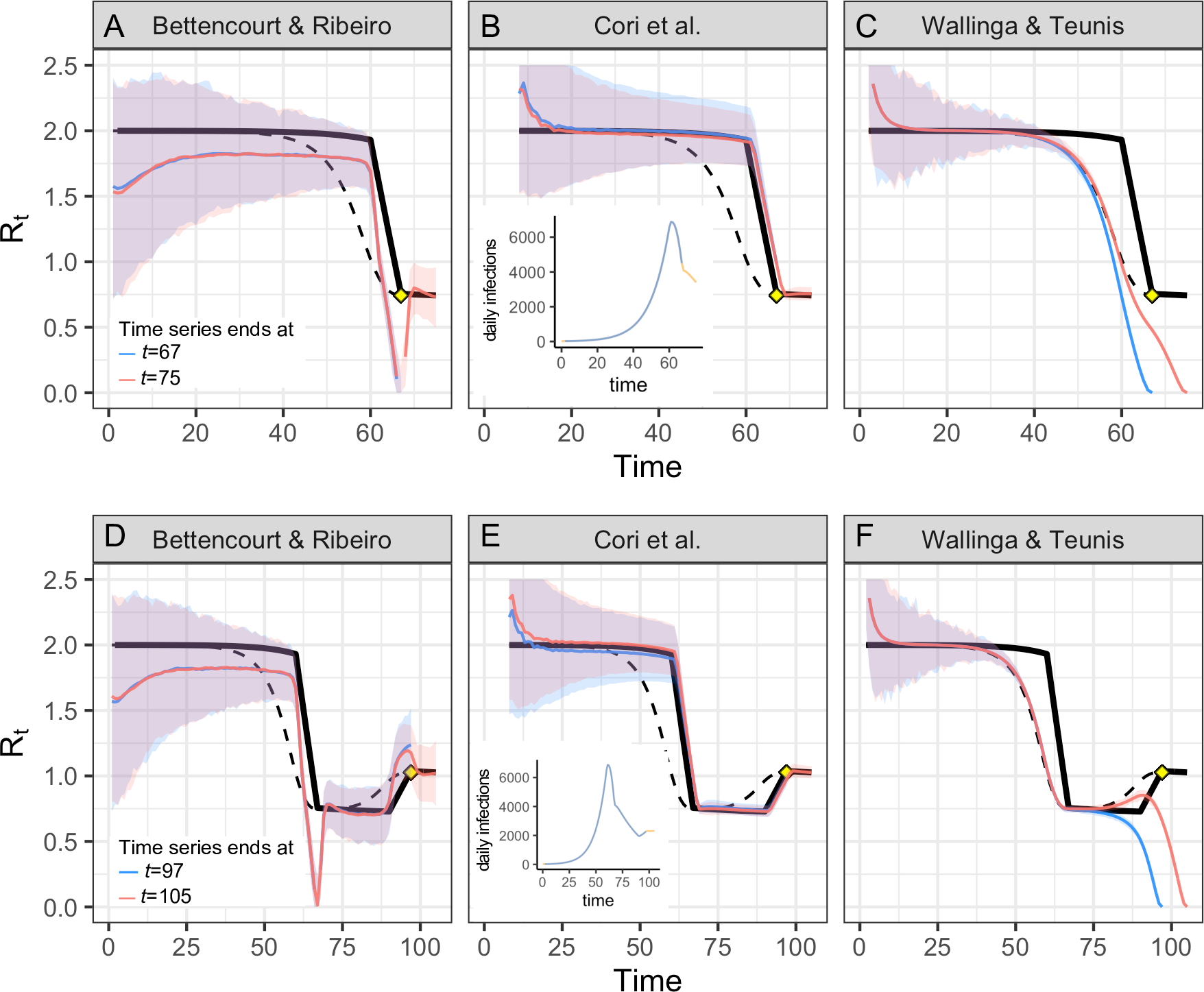

Supplement: S1 Fig — (A–C) Alternate version of Fig 2 in which the time series ends on the day Rt first hits its minimum value after falling abruptly (time 67, yellow point) or 8 days after the changepoint (time 75). (D–F) The time series ends on the day Rt stops rising (time 97, yellow point) or 8 days later (time 105). Estimates of the instantaneous reproductive number (A, B, D, E) remain accurate to the end of the time series, and estimates do not change as new observations become available in the 8 days following the changepoint. As in the main text, estimates of the unadjusted case reproductive number (C, F) depend on data from not-yet-observed time points. These estimates become more accurate as new observations are added to the end of the time series (orange vs. blue). Methods to infer the number of not-yet-observed infections can help make estimates of the case reproductive number more accurate in real time [4, 29]. All panels show fits to the time series of new infections and assume all infections are observed instantaneously. Solid black line shows the instantaneous reproductive number, and dashed black line shows the case reproductive number. Colored lines and confidence region show posterior mean and 95% credible interval (A, B, D, E) or maximum likelihood estimate and 95% confidence interval (C, F). (TIF) [file pcbi.1008409.s001.tif]

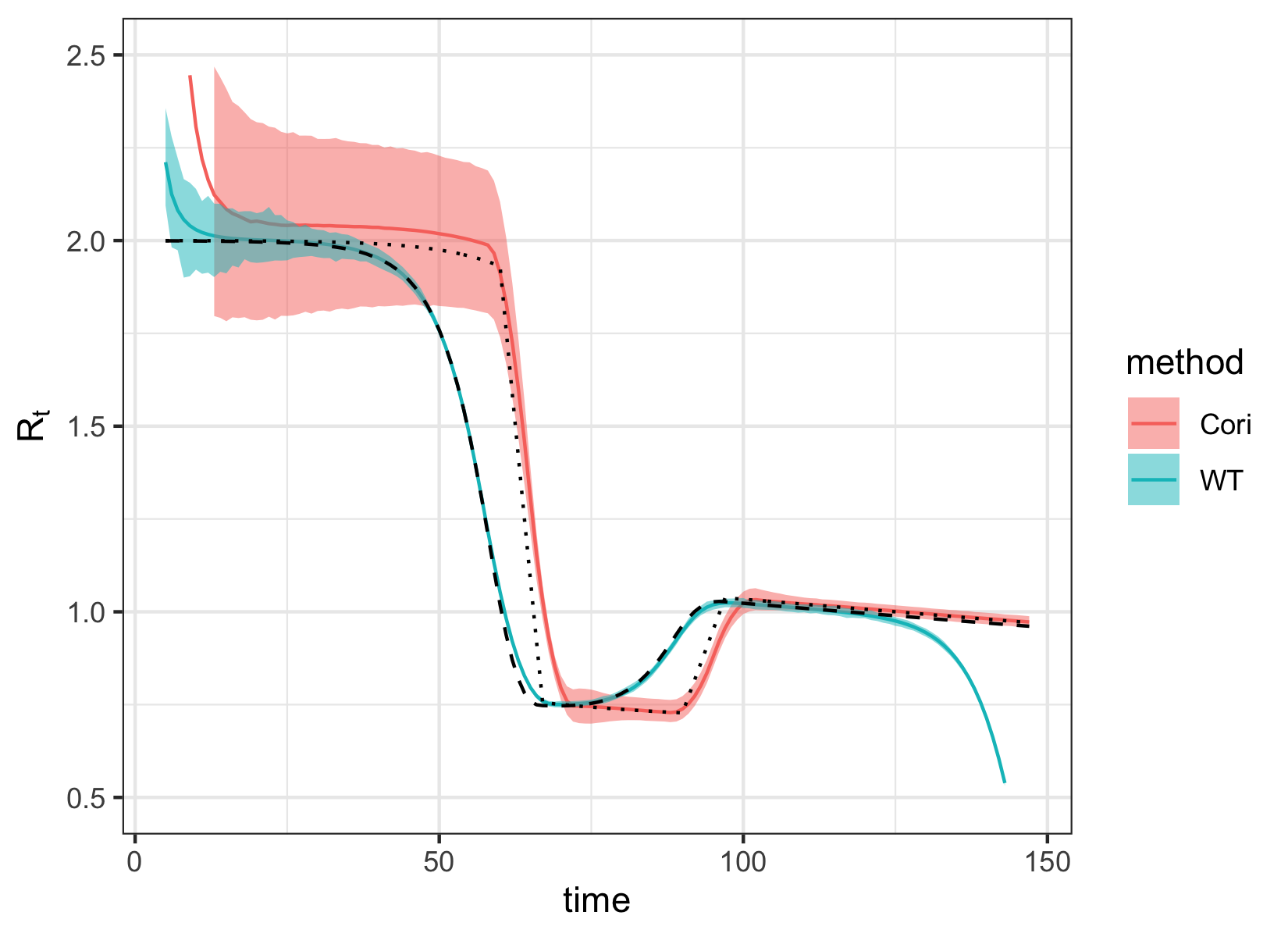

Supplement: S2 Fig — Both were estimated using a 7-day smoothing window on a synthetic time series of new infections, observed without delay. The estimates of Cori et al. and Wallinga and Teunis are similar in shape when smoothed, but the estimate of Wallinga and Teunis (the case reproductive number) leads that of Cori et al. (the instantaneous reproductive number) by roughly 8 days, or the mean generation interval. Solid colored lines and confidence regions show the posterior mean and 95% credible interval (Cori et al.) or maximum likelihood estimate and 95% confidence interval (Wallinga and Teunis). Dotted and dashed lines show the exact instantaneous reproductive number and case reproductive number, respectively. (TIF) [file pcbi.1008409.s002.tif]

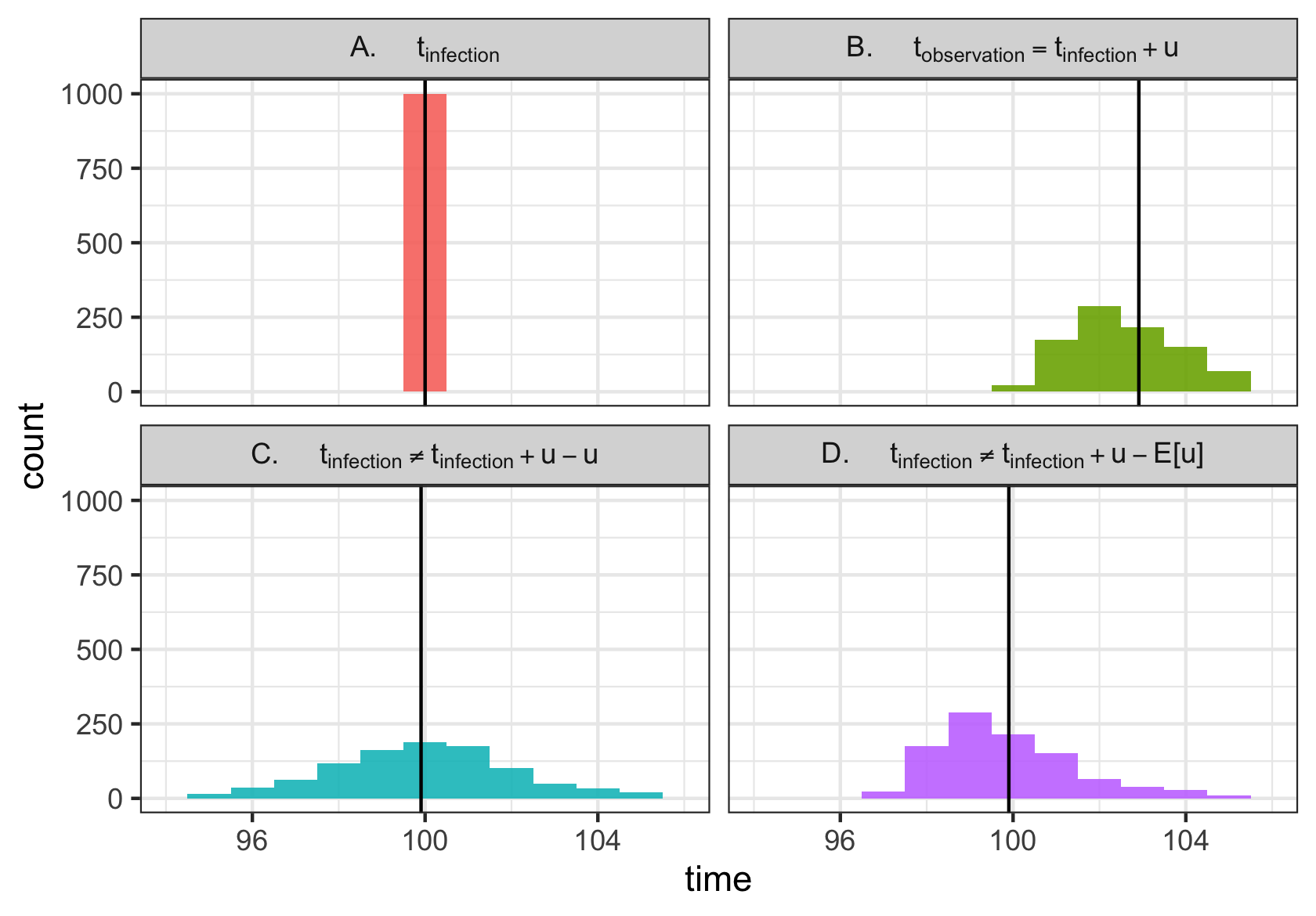

Supplement: S3 Fig — (A) Consider 1,000 individuals all infected at time 100 (vertical line shows the mean). (B) Now consider the times at which these individuals are observed. Logically, tobservation = tinfected+u, where u is a random variable describing the delay between infection and observation. Mathematically, this is a convolution of the infection time and the delay distribution. Because u has non-zero variance, observation times are not only shifted into the future but also blurred across many dates. This blurring is biologically realistic; due to the variability in disease progression and care seeking, individuals with the same date of infection will not necessarily be observed at the same time. (C) Using the observations in panel B, we aim to recover the latent times of infection shown in panel A. Doing so would require not only shifting into the past but also removing the variance introduced by the observation process, which can be achieved by deconvolution. Instead, as demonstrated here, a common strategy is to subtract u from the times of observation, effectively repeating the convolution shown in panel B, but this time moving backward in time rather than forward. This is not the correct inverse operation. It fails to remove variance introduced by the observation process (the forward convolution) and adds new biologically unrealistic variance, further blurring the inferred times of infection. (D) Shifting the times of observation by the mean delay E[u] is also incorrect, as it does not remove the variance from the forward convolution in panel B. But if the mean delay time is known exactly, this approach is preferable to panel C, as it avoids adding even more variance. Ultimately, deconvolution methods would be needed to recover panel A from the observations in panel B while properly accounting for uncertainty. (TIF) [file pcbi.1008409.s003.tif]
